# Comparative Analysis of Sucrose-Regulatory Genes in High- and Low-Sucrose Sister Clones of Sugarcane

**DOI:** 10.3390/plants13050707

**Published:** 2024-03-01

**Authors:** Qaisar Khan, Ying Qin, Dao-Jun Guo, Yu-Yan Huang, Li-Tao Yang, Qiang Liang, Xiu-Peng Song, Yong-Xiu Xing, Yang-Rui Li

**Affiliations:** 1 Guangxi Key Laboratory of Sugarcane, College of Agriculture, Guangxi University, Nanning 530004, China; qaisar.khan@yahoo.com (Q.K.); yingqinemail@163.com (Y.Q.); gdj0506@163.com (D.-J.G.); yygxu1028@163.com (Y.-Y.H.); liyr@gxu.edu.cn (L.-T.Y.); 2 Guangxi Key Laboratory of Sugarcane Genetic Improvement, Key Laboratory of Sugarcane Biotechnology and Genetic Improvement (Guangxi), Ministry of Agriculture and Rural Affairs, Sugarcane Research Institute of Guangxi Academy of Agricultural Sciences, Nanning 530003, China; liangqiangde@163.com

**Keywords:** sucrose phosphate synthase, sucrose phosphate phosphatase, sucrose synthase, cell wall invertase, neutral invertase, reducing sugar, non-reducing sugar, RT-qPCR

## Abstract

Sugarcane is a significant primitive source of sugar and energy worldwide. The progress in enhancing the sugar content in sugarcane cultivars remains limited due to an insufficient understanding of specific genes related to sucrose production. The present investigation examined the enzyme activities, levels of reducing and non-reducing sugars, and transcript expression using RT-qPCR to assess the gene expression associated with sucrose metabolism in a high-sucrose sugarcane clone (GXB9) in comparison to a low-sucrose sister clone (B9). Sucrose phosphate synthase (SPS), sucrose phosphate phosphatase (SPP), sucrose synthase (SuSy), cell wall invertase (CWI), soluble acid invertase (SAI), and neutral invertase (NI) are essential enzymes involved in sucrose metabolism in sugarcane. The activities of these enzymes were comparatively quantified and analyzed in immature and maturing internodes of the high- and low-sucrose clones. The results showed that the higher-sucrose-accumulating clone had greater sucrose concentrations than the low-sucrose-accumulating clone; however, maturing internodes had higher sucrose levels than immature internodes in both clones. Hexose concentrations were higher in immature internodes than in maturing internodes for both clones. The SPS and SPP enzymes activities were higher in the high-sucrose-storing clone than in the low-sucrose clone. SuSy activity was higher in the low-sucrose clone than in the high-sucrose clone; further, the degree of SuSy activity was higher in immature internodes than in maturing internodes for both clones. The *SPS* gene expression was considerably higher in mature internodes of the high-sucrose clones than the low-sucrose clone. Conversely, the *SuSy* gene exhibited up-regulated expression in the low-sucrose clone. The enhanced expression of *SPS* in the high-sucrose clone compared to the low-sucrose clone suggests that *SPS* plays a major role in the increased accumulation of sucrose. These findings provide the opportunity to improve sugarcane cultivars by regulating the activity of genes related to sucrose metabolism using transgenic techniques.

## 1. Introduction

Sugarcane (*Saccharum officinarum* L.) is a very valuable crop used for the manufacture of sugar and bioethanol [1]. The amount and quality of the soluble sugar, mainly sucrose, directly affect the sugar yield in sugarcane [2]. Sugarcane is a major source of sucrose, which is used as the principal source of sugar in human consumption, animal feed, and biofuel generation [3,4]. In addition, sugarcane is a crucial crop for the worldwide economy due to its production of many byproducts, such as bagasse, bagasse ash, press mud, molasses, vinasse, alcohol, yeast, wasted wash, and other derivatives [5,6]. Physiological changes occur sequentially in different internodes of the sugarcane stalk, including immature, maturing, and mature internodes located at the top, middle, and bottom. Immature internodes of sugarcane stalks are composed of fibrous tissue containing a significant amount of sugar that can be reduced while having a low quantity of sucrose. As the internodes mature, the growth rate gradually decreases until maturity is achieved [7,8,9].

The escalating emissions of greenhouse gases (GHGs) and subsequent global warming because of climate change have led to a rise in the incidence and harshness of immoderate weather phenomena [10]. The extent of the influence of climate change on sugarcane is determined by the geographical position and the ability to adapt to it. Climate unevenness and variation are expected to cause alterations in sea levels, precipitation patterns, and the occurrence of exceptionally hot and cold events, floods, droughts, and other non-living environmental pressures, including tornadoes and hurricanes [11,12,13]. These variables greatly impede the growth and development of sugarcane plants [14]. Sugarcane plants respond to varied environmental factors by employing multiple mechanisms and generating different metabolites, including antioxidants, osmoprotectants, and heat shock proteins, along with activating distinct metabolic processes. One area of scientific inquiry that lacks a comprehensive understanding is the process of sugarcane maturity and the impact of diverse cultivars, crop growth stage, and climate on this process. Sugarcane maturity refers to the process of sucrose accumulation in the stalks, which is significantly impacted by several circumstances [15]. To a certain extent, research has been carried out on the process of sugarcane maturation [16]; however, the consequence of an intricate array of environmental variables, the genetic capacity of varieties and crop management, soil moisture, ambient temperature, and other ecological factors interacting with sugarcane are the primary areas involved in sugarcane ripening [17], which still needs further investigation. The amalgamation of these factors induces the pace of growth, yield, ripening process, and sucrose accumulations, so additional scrutiny is required to determine the pinpoint role of each factor [18].

Generally, plants are split into “source” where carbon dioxide fixation takes place, and “sink” where excess assimilates are stored [19]. In sugarcane, leaves are classified as “source” due to their role in fixing carbon dioxide through photosynthesis [20]. In contrast, stalks are regarded as “sink” where excess sugar is stored [21]. The quantity of sucrose originating from the source is contingent upon the photosynthetic activity and the allocation of carbon among different activities, such as the synthesis of starch in the chloroplast [22] and the transfer of triose-phosphates from the chloroplast. These processes control the production and buildup of sucrose in the vacuole. An alteration in these regulators affects the connection between sources and sinks in sugarcane. The source–sink relationship is an intricate phenomenon that requires thorough clarification. Nevertheless, it is widely recognized that the ultimate location for storing sucrose is the parenchyma cells found in the stalk of sugarcane, rather than the sink tissues [23,24,25].

The main goal of commercial sugarcane cultivation is the sucrose content. Sugar transporters help the phloem carry sucrose from the leaves, where photosynthesis produces it, to the internodes [26,27,28]. After that, it can either be broken down into glucose and fructose to supply the energy needed for development and growth or stored in the internodes [29]. While growth and development decrease the sucrose content in internodes, they also allow the plant factory to enhance sucrose production and store more sucrose in the storage sink [30]. The grand growth phase is of utmost importance for the accumulation of sucrose, as it is during this period that a balance is achieved for sucrose production, growth use, and storage in the sink [31]. During the maturation phase of the cane, any extra fructose, glucose, and other soluble sugars are transformed into sucrose and deposited in sinks for accumulation [32].

Multiple genes are involved in the production, breakdown, and storage of sucrose in sugarcane; for example, *sucrose phosphate synthase* (*SPS*), *sucrose-phosphate phosphatase* (*SPP*), *sucrose synthase* (*SuSy*), *β-fructofuranosidase*, *soluble acid invertase* (*SAI*), *acid invertase* (*AI*), *neutral invertase* (*NI*), and *cell wall invertase* (*CWI*) have been identified in the apoplast and cell walls of sugarcane [33]. Vacuoles contain soluble *acid invertase (SAI)* [34], which primarily operates in rapidly developing tissues such as immature internodal tissue, root apices, and cell and tissue culture. Upon entering parenchyma cells, hexoses undergo a transformation into sucrose, facilitated by the enzymes SPS and SPP [35]. The sucrose level varies between high- and low-sugar sugarcane genotypes and between immature and mature internodes. Recent advancements in sugarcane breeding have resulted in notable enhancements in agronomic features [36,37]. However, further research is required to fully understand the intricate mechanism of sucrose metabolism in sugarcane [9,38,39]. An essential target for future studies is to investigate the genes linked to sugar synthesis, sucrose metabolism, and accumulation in internodes of sugarcane with variation in age.

SuSy is associated with sucrose metabolism in plant sinks, where it catalyzes the production of uridine diphosphate glucose or adenosine diphosphate glucose and fructose from the decomposition of sucrose [40]. SuSy also contributes to plant growth and development, particularly in the shoot apical meristem [41,42,43]. SuSy activities positively influence glucose and fructose concentrations, while negatively affecting sucrose accumulation in sugarcane. The genotypes with high SuSy expression had low sucrose levels but greater hexose levels, and vice versa. SuSy may provide hexose sugars for glycolysis in fast-growing cells and be the precursor to other growth progressions [44]. The breakdown of sucrose into hexoses is required for the extraction of carbon and energy [45,46]. SuSy participates in physiological activities in plants, including the production or breakdown of sucrose in different sinks. However, SuSy appears to play a function in sucrose breakdown rather than in production, primarily in young developing tissues [40,47].

The present work aimed to examine the regulation mechanism of sucrose metabolism by comparing the immature and maturing internodes of a high-sucrose sugarcane clone “GXB9” with those of a low-sucrose clone “B9” after 250 days of planting (DAP), using RT-qPCR. Additionally, tests were conducted to measure the activities of enzymes and the concentration of sugars. The objective of the study was to compare the expression of genes associated with sucrose accumulation and metabolism regulation, including SPS, SPP, SuSy, SAI, CWI, and NI, by RT-qPCR analysis in the immature and maturing internodes of both clones. To the best of our knowledge, this is the first study to analyze the GXB9 and B9 sugarcane clones comparatively for sucrose metabolism.

## 2. Results

In this study, the activities of SPS, SPP, SAI, NI, CWI, and SuSy in the high-sucrose clones GXB9 and the low-sucrose clones B9 were compared, and the transcript expression in immature and maturing internodes was analyzed by RT-qPCR.

### 2.1. Reducing and Non-Reducing Sugar Concentrations

**Figure 1:**
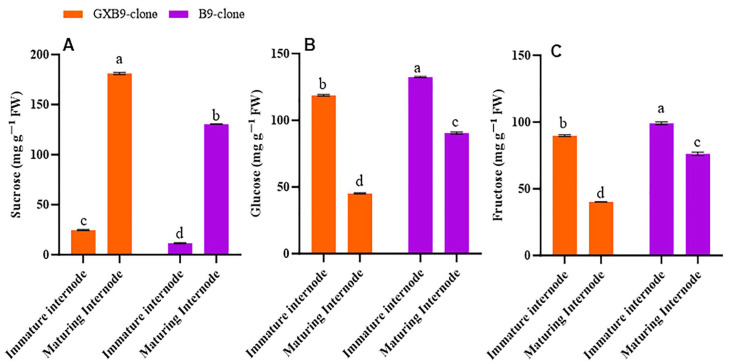
Figure 1 illustrates differences in (**A**) sucrose, (**B**) glucose, and (**C**) fructose concentrations between high- (GXB9) and low-sucrose (B9) sister clones of sugarcane and immature and maturing internodes of identical clones. The figure legends GXB9 and B9 represent sugarcane sister clones. The *x*-axis represents the different types of internodes, while the *y*-axis represents the concentration of sugars. Different lowercase letters on bars show a significant difference, at *p* < 0.05, among both internode types and sister clones using the. The data have been presented as mean ± SE (standard error). The unit “mg g^−1^ FW” is milligram per gram of fresh weight.

Disparities in sucrose and sugar concentrations were observed between the sister clones that accumulate high and low amounts of sucrose. Furthermore, there were variations in the sucrose and hexose levels between the immature and maturing internodes of the same clone. The sucrose concentration in immature internodes was lower than in the maturing internodes, while the contents of glucose and fructose were higher in immature internodes compared to the maturing internodes for the high-sucrose clone. Immature internodes have lower sucrose concentrations, and maturing internodes have high sucrose concentrations in the low-sucrose clones. However, glucose and fructose concentrations were more significant in immature internodes than in maturing internodes in the low-sucrose-accumulating clone. For both clones, sucrose concentration was higher in maturing internodes, while glucose and fructose concentrations were higher in immature internodes. Overall, the high-sucrose-accumulating clone had higher sucrose concentrations than the low-sucrose-accumulating sister clone (Figure 1).

### 2.2. Enzymes Activities Level

**Figure 2:**
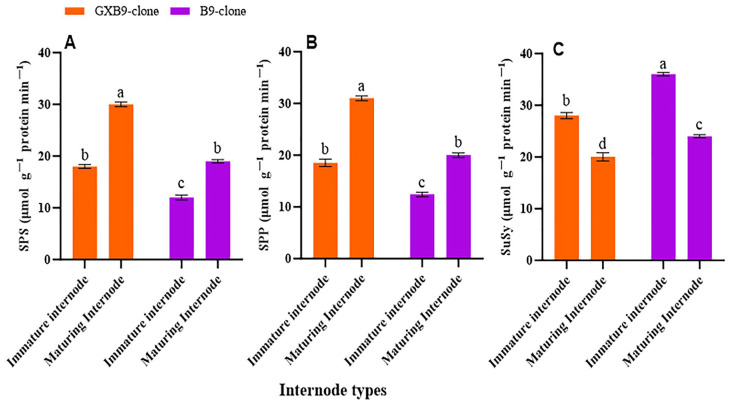
Shows variation in activities of (**A**) sucrose phosphate synthase (SPS), (**B**) sucrose phosphate phosphatase (SPP), and (**C**) sucrose synthase (SuSy) between high (GXB9) and low (B9) sucrose sister clones of sugarcane and immature and maturing internodes of identical clones. The figure legends GXB9 and B9 represent sugarcane sister clones. The *x*-axis denotes the different types of internodes, whereas the *y*-axis represents the magnitude of enzyme activities. Different lowercase letters on bars show a significant difference, at *p* < 0.05, among both internode types and sister clones using the least significant difference (LSD) test. The data have been presented as mean ± SE (Standard error). The unit “µmol g^−1^ protein min^−1^” is micromole per gram of protein per minute.

**Figure 3:**
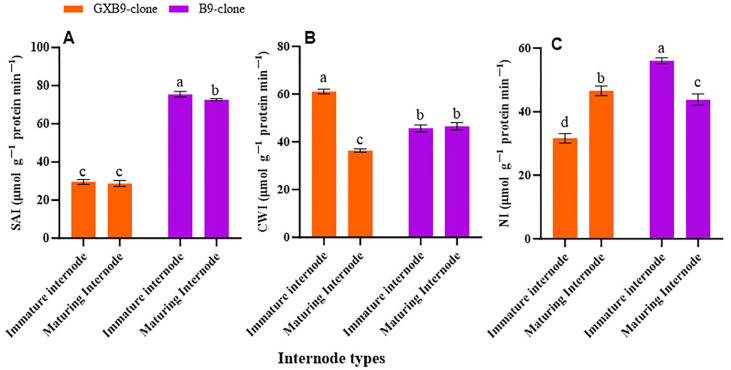
Figure 3 displays disparity in activities of (**A**) soluble acid invertase (SAI), (**B**) cell wall invertase (CWI), and (**C**) neutral invertase (NI) between high- (GXB9) and low-sucrose (B9) sister clones of sugarcane and immature and maturing internodes of identical clones. The figure legends GXB9 and B9 represent sugarcane sister clones. The *x*-axis denotes the different types of internodes, whereas the *y*-axis represents the magnitude of enzyme activities. Different lowercase letters on bars show a significant difference, at *p* < 0.05, among both internode types and sister clones using the least significant difference (LSD) test. The data have been presented as mean ± SE (standard error). The unit “µmol g^−1^ protein min^−1^” is micromole per gram of protein per minute.

The activities of SPS, SPP, SuSy, SAI, CWI, and NI in the two sister sugarcane clones were assessed and subjected to comparative analysis. The activities of the SPS and SPP were greater in the clone with greater sucrose accumulation compared to that with lower sucrose accumulation. The SPS and SPP activities were shown to be higher in mature internodes of the high-sucrose clone compared to immature internodes of the same clone. The activity of SuSy was higher in the clone with lower sucrose accumulation compared to that with higher sucrose accumulation. The low-sucrose clone exhibited higher SuSy activity in immature internodes compared to matured internodes. The participation of SuSy was shown to be higher in immature internodes, regardless of the sucrose concentration in both clones. Conversely, the activity of SPS was seen to be higher in the maturing internodes of both clones (Figure 2). SAI, CWI, and NI activities were greater in immature internodes of the low-sucrose clone than in the same internodes of the high-sucrose clone. In contrast, the activity levels of CWI and NI were higher in maturing internodes of the high-sucrose clone compared to the low-sucrose clone. The SAI activity was higher in maturing internodes of the low-sucrose clone in contrast to those of the high-sucrose clone (Figure 3).

### 2.3. RT-qPCR Data Analysis

**Figure 4:**
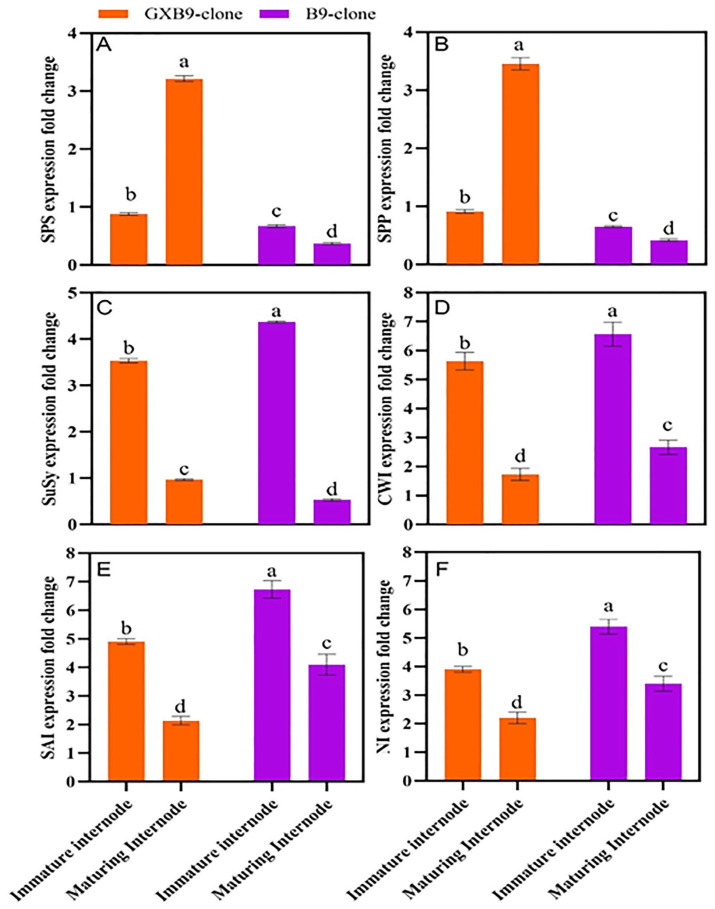
Figure 4 indicates differential expression regulation of sucrose-metabolism-related genes (**A**) *sucrose phosphate synthase* (*SPS*), (**B**) *sucrose phosphate phosphatase* (*SPP*), (**C**) *sucrose synthase* (*SuSy*), (**D**) *cell wall invertase* (*CW*I), (**E**) *soluble acid invertase* (*SAI*), and (**F**) *neutral invertase* (*NI*), between high- (GXB9) and low-sucrose (B9) sister clones of sugarcane and immature and maturing internodes of identical clones. The figure legends GXB9 and B9 represent sugarcane sister clones. The *x*-axis denotes the different types of internodes, while the *y*-axis shows the fold change in values of gene expression. Different lowercase letters on bars show a significant difference, at *p* < 0.05, among both internode types and sister clones using the least significant difference (LSD) test. The data have been presented as mean ± SE (standard error). Genes with a fold change (FC) value ≥ 1 were up-regulated, whereas genes with a fold change value ≤ 1 were down-regulated.

The analysis of RT-qPCR data derived from the pools of immature and ripening internode samples of high- and low-sucrose sugarcane clones revealed varying levels of gene expression in different clones and internodes (Figure 4). The *SPS*, *SPP*, *SuSy*, *CWI*, *SAI*, and *NI* genes were selected for assessing the relative expression in the high-sucrose clone “GXB9” and low-sucrose clone “B9”. Both clones exhibited expression of the selected genes, and the *SPS* gene was up-regulated in the clone with a higher sucrose level and down-regulated in the clone with a lower sucrose level. The *SPS* gene exhibited notable differences in expression level between immature and maturing internodes of the high-sucrose clone. Specifically, it was up-regulated in maturing internodes and down-regulated in immature internodes. The *SPP* gene was down-regulated in immature internodes of both clones; however, it was up-regulated in maturing internodes of the high-sucrose clone but down-regulated in the low-sucrose clone. The *SuSy* gene exhibited a higher expression level in immature internodes compared to maturing internodes in both clones. The higher expression of the *SPS* gene in the maturing internode of the high-sucrose clone compared to the low-sucrose clone suggests its significant contribution to the increased sucrose accumulation. *CWI*, *SAI*, and *NI* genes were up-regulated in immature and maturing internodes of both clones; however, immature internodes showed higher expression levels compared to maturing internodes.

## 3. Discussion

Sugarcane is a significant agricultural commodity responsible for around 80 percent of the world’s sugar production. China ranks fourth among the major sugar-producing nations [48]. RT-qPCR is a crucial tool for determining the expression of target genes in samples of interest [49]. The primary metabolic pathways for sucrose in the internodes of sugarcane involve SPS, SPP, SuSy [50], SAI, NI, and CWI [51]. Sucrose synthesis in green leaves and transport to storage and consuming tissues are co-ordinated appropriately in sugarcane. Within the parenchyma cells of internodal tissues, the sugars undergo metabolic processes and are, subsequently, resynthesized through the actions of SPS and SuSy [52,53].

### 3.1. Environmental Interaction and Sucrose Improvement

Climate change poses a threat to sugarcane both directly, through variations in temperature and precipitation, and indirectly, through shifting the intensity of other biotic and abiotic factors, an abundance of pollutants, and the functioning of other ecosystem actors that impact sugarcane productivity [11,54]. A complex interplay between climate factors, cultivar genetic potential, and agricultural management determines when sugarcane ripens. Ripening is a natural process of sucrose accumulation acting as a shield against unfavorable environmental conditions, which is not the same as the maturity of the stalk [55]. Sugarcane crops have numerous difficulties in terms of adjusting of varieties to various production environments, with an emphasis on choosing desired traits in accordance with environmental circumstances [56]. Technological criteria, which include the percentages of moisture, polarizable sugar, fiber, and apparent sucrose and soluble solids in the juice, as well as other intrinsic features of the plant, can determine the quality of sugarcane used to produce both sugar and ethanol [57]. Therefore, it is suggested that future research based on characteristics of importance to sugarcane agro-industries for high sugarcane yields to gain sustainability should concentrate on identifying potential sugarcane varieties for increased sucrose production in variable environmental conditions.

### 3.2. Free Sugars

The current study discovered the differences in the sucrose and sugar storage between immature and maturing internodes of identical clones and between the low- and high-sucrose-accumulating sister clones. Compared to the low-sucrose clone, the high-sucrose-accumulating clone had a greater sucrose concentration. Higher glucose and fructose concentrations were found in immature internodal tissues compared to maturing internodes, whereas lower sucrose contents were found in the latter. Our results are in line with the findings of other studies that were published [16,58,59]; that is, immature internodes had high hexoses and little sucrose, while maturing internodes had the opposite pattern. The current study also observed that the activity of the SPS enzyme was greater in the clone with a higher sucrose accumulation than in that with a lower sucrose level. Maturing internodes exhibited superior SPS performance compared to immature ones. The presence of SuSy activity was observed to be higher in immature internodes compared to maturing internodes. The observed disparities in the levels of reducing and non-reducing sugars and enzyme activity suggest that the GXB9 clone may have undergone genetic mutations, leading to the observed variations despite both clones having identical genetic backgrounds. However, further experimental verification is still required.

### 3.3. Invertase Participation in Sucrose Metabolism

Invertase is the primary enzyme responsible for the breakdown of sucrose into glucose and fructose, which serves as an energy source for cellular development, elongation, and various metabolic activities [60,61]. SAI is thought to play a significant role in controlling hexose levels in specific tissues and is concerned with the rate at which sugar returning from storage. There is a noticeable seasonal variance in SAI activity, which is strong during a period of rapid growth and less in mature tissues [62]. The SAI enzyme potentially contributes to the release of stored sucrose from the vacuole, and its activity level was particularly elevated during the elongation phase [63]. In our finding, the SAI activity was higher in the immature internode of both clones as compared to the maturing internode, which is consistent with the findings of the following researchers. The increased SAI activity observed in the immature internode, as compared to the maturing internode, for both clones aligns with the findings of [64], who reported a higher SAI activity in the immature internode compared to the maturing internode. The activity of SAI reduces as internodes mature, suggesting a correlation between the decrease in SAI activity and the maturation of internodes [65].

It is commonly believed that cell wall invertase allows sucrose to enter cells in developing tissues via phloem loading [66]. Sugarcane top internodes had increased cell wall invertase expression, which decreased significantly with maturity and was very low in mature internodes [67]. In our results, the activity of CWI was more significant in immature internodes than in maturing internodes of both clones; however, maturing internodes of the low-sucrose clone have higher CWI activity than the high-sucrose clone, which is in alignment with the above-reported results.

Ebrahim et al. [68] and Vorster et al. [69] found that the activity of NI increases in developing internodes and decreases in maturing internodes. Additionally, it is shown that NI is the sole enzyme responsible for breaking down sucrose and is directly associated with sugar levels in fully developed sugarcane internodal tissue, where a positive correlation between NI activity levels and hexose concentrations in the respective tissues is found reported by Gayler et al. [70] and Bosch et al. [71], and a negative correlation between NI activity levels and sucrose quantity has been noticed by Rose and Botha [72]. In the current study, it was found that NI activity was higher in immature internodes than maturing internodes of both low- and high-sucrose sugarcane clones. Overall immature and maturing internodes of the low-sucrose clone showed higher NI activity than the high-sucrose one. These findings agree with earlier reports cited here stating that NI activities are higher in maturing internodes than mature internodes of sugarcane.

### 3.4. Predominant Role of SPS Gene in Sucrose Accretion

*SPS* is the primary gene required for plant sucrose production and control [73,74,75]. Previous research has found that the *SPS* gene is expressed more intensely in maturing internodes of sugarcane than in immature internodes [76,77,78]. However, an earlier report on *SPS* expression showed that *SPS* activity is higher in immature internodes than in mature internodes [79]. *SPS* gene activity has been found to be higher in high-sucrose sugarcane growing tissues than in low-sucrose tissues [77]. The SPS activity declines with the sugarcane plant’s maturity [67,80]. It transforms fructose-6-phosphate and uridine diphosphate-glucose into sucrose-6-phosphate, an important precursor of sucrose synthesis [81]. The current study revealed a significant up-regulation of the *SPS* and *SPP* genes in high-sucrose sugarcane clones compared to low-sucrose clones. Moreover, *SPS* expression was higher in maturing internodes than immature ones of the high-sucrose sugarcane clone. Thus, the up-regulation of the *SPS* gene in the high-sucrose clone and maturing internodes shows that it plays a dominating role in synthesizing and accumulating increased sucrose content in high-sucrose sugarcane clones.

### 3.5. Role of SuSy Gene in Sucrose Metabolism

The current investigation showed that the *SuSy* gene was more highly expressed in the lower-sucrose clone than in the higher-sucrose clone, and it was more abundant in immature internodes than maturing internodes, demonstrating its importance in sugarcane-growing tissues via sucrose lysis. As a result, internodes with higher *SuSy* activity have lower sucrose concentrations. The current study’s findings about the *SuSy* gene are consistent with prior findings cited here [40,44,47]. The expression of *CWI*, *SAI,* and *NI* genes were higher in immature internodes than in maturing internodes of both high- and low-sucrose sister clones. Finally, the expression level of invertase genes was comparatively greater in the low-sucrose clone compared to the high-sucrose clone, which support the results of relevant enzyme activities.

## 4. Materials and Methods

### 4.1. Experimental Material and Sample Collection

The Guangxi Academy of Agriculture Science (GAAS) in Nanning, China, provided the sugarcane clone GXB9, which contains high sucrose, and B9, which has low-sucrose content, for experimental use. The “B9” clone is known for its low sugar content and great yield, as well as its remarkable development and growth [82]. B9 and GXB9 clones share the same genetic lineage, but GXB9 has a higher sugar content than B9. In October 2013, the high-sugar-content clone was identified among the population of the low-sugar clone B9 (parent) during the regular sugar-testing procedure [83]. The recently identified clone with elevated sugar levels was designated as Guixuan B9 (GXB9). Over the course of several years, both clones underwent testing at various field stations to determine any disparities in sugar concentration. The findings continually revealed a notable difference in sugar content between B9 and GXB9 [84].

On 7 March 2022, sugarcane setts were planted at the experimental field of the College of Agriculture, Guangxi University in Nanning, China. The cultivation of sugarcane followed all normal standards, including regular irrigation, weed removal, disease inspection, fertilization (N-P_2_O_5_-K_2_O: 22-8-12) (Kingenta Ecological Engineering Group Co., Ltd., Binzhou, China), metsulfuron herbicides (Shandong Qiaochang Chemical Co., Ltd., Binzhou, China), and carbendazim fungicide (Jiangyin Fuda Agrochemical Co., Ltd., Jiangyin, China) [85].

A total of six sugarcane plants were selected at random from each plot. Samples from the upper immature (1–4) and middle maturing (12–15) internodes of both clones were obtained in mid-November, 250 DAP, for the purpose of analyzing enzymes activities and transcript expression, as well as reducing and non-reducing sugars. The samples were obtained during the early morning hours (8:00–9:00 a.m.) and promptly immersed in liquid nitrogen to halt the metabolic processes related to sucrose and sugar levels. The samples were preserved at a temperature of −80 °C for subsequent study.

### 4.2. Reducing and Non-Reducing Sugar Extraction and Analysis

Samples were prepared by removing the rind of the fresh immature and maturing internodes collected from high- and low-sucrose clones GXB9 and B9, and soft tissue were subsequently diced into minute fragments and combined. These prepared basic samples were stored at −80 °C and used in the upcoming analysis according to the purpose.

Sucrose, glucose, and fructose were obtained from a 15 g sample of immature and maturing internodes of low- and high-sucrose clones, respectively. The weighed samples were subjected to boiling in 85% ethanol and, subsequently, in 75% ethanol for durations of 18 and 20 min, respectively. The extract was transformed into syrup by submitting it to a process of reduced pressure at a temperature of 40 °C. The presence of sucrose was identified using the approach described by the method described by Roe [86] with modification. The sucrose concentration was determined using a 1 mL mixture consisting of 500 µL of extract and 500 µL of a 6% KOH solution. The tubes containing the reaction mixture were immersed in an 80 °C water bath for 22 min, and then cooled to ambient temperature. The cooled reaction mixture was supplemented with 12 mL of resorcinol (0.2%) and 5 mL of HCl (32%), and thereafter subjected to incubation at 90 °C for 15 min. The sucrose concentration was determined at a wavelength of 540 nm using Shimadzu UV 1600 Double Beam Spectrophotometer Kyoto, Japan, and then compared to a standard curve. The hexose concentrations in the samples were measured using the methodology described by Somogyi [87] with some amendments. In summary, 120 µL of samples were collected in a test tube, and the volume was adjusted to 2.5 mL by adding distilled water. Next, 1.5 mL of alkaline tartrate reagent was added, and the mixture was subjected to incubation in a boiling bath for 12 min. The samples were chilled to ambient temperature, and then 1.2 mL of arsenomolybdic reagent was added. After incubating at room temperature for 15 min, the reaction volume was adjusted to 12 mL using distilled water, and the absorbance was measured at 620 nm using Shimadzu UV 1600 Double Beam Spectrophotometer, Kyoto, Japan. The quantity of reducing sugars was determined with the help of a glucose standard curve.

### 4.3. Enzyme Isolation

The internodal tissue samples that were prepared in advance and stored at −80 °C were used for the extraction of enzymes, including SPS, SPP, SuSy, CWI, SAI, and NI, according to the related methodologies.

The enzymes were extracted from 350 g of diced internodal tissues by homogenizing them at 5 °C. The homogenization was carried out using a pH 7.4 Tris-HCl (0.12 M) buffer containing TritonX-100 (1.5%), cysteine-HCl (0.03 M), MgCl_2_ (0.02 M), EDTA (0.03 M), DIECA (0.03 M), and mannitol (0.35 M). Prior to usage, the buffer was supplemented with complete protease inhibitor cocktail tablets (Takara Biomedical Technology Co., Ltd., Beijing, China) according to the manufacturer’s instructions. The homogenate was cleaned from debris using a double nylon filter, resulting in the collection of the filtrate. The extraction process was iterated for the residue using the same buffer. The residue was then filtered through a nylon filter in a similar manner and, subsequently, washed with more buffer. Afterward, the liquid that passed through the filter was centrifuged at a speed of 14000 rpm for 12 min. The pellet was resuspended in buffer, and centrifugation was repeated. Three different concentrations of (NH4)_2_SO_4_, that is, 0–30%, 30–60%, and 60–80%, were added to the mutual supernatant of the enzymes. This resulted in the formation of three different fractions of precipitate. It is worth noting that no decrease in enzyme activity was observed. The fraction of precipitate obtained at 30–60% (NH4)_2_SO_4_ was selected for enzyme analysis due to the optimal activity of both enzymes seen within this concentration of (NH4)_2_SO_4_. The chosen precipitate was fragmented and, subsequently, dissolved in a small volume of extraction buffer. The solubilized precipitate was subjected to dialysis against the solvent buffer by placing it for 24 h at a temperature of 5 °C. Subsequently, a solution containing 22% glycerol was added to the dialysate. The mixture was then dipped in liquid nitrogen and stored at a temperature of −80 °C for subsequent analysis.

### 4.4. Enzymes Assay

The functional level of SPS and SPP were measured in a total volume of 1 mL containing 200 µL Tris-HCl (1.2 M, pH 8) composed of MgCl_2_ (1.1 M), sodium fluoride (NaF, 2.2 mM), 200 µL UDPG (4.2 mM), and 200 µL fructose-6-phosphate (8.2 mM), and 400 µL dialyzed preparation having 22–24 µg protein µL^−1^. The reaction mixture was incubated at a temperature of 37 °C for 30 min. The reaction was terminated by adding 200 µL NaOH (1.0 N). The reaction mixture was immersed in the water bath for 15 min. Subsequently, 300 µL of resorcinol solution (0.15%, dissolved in 90% ethanol) and 800 µL 28% hydrochloric acid (HCl) were added to the reaction mixture. The combination underwent incubation at 80 °C for 12 min. Then, the reaction mixture was allowed to cool to ambient temperature, and the optical density of the reaction mixture was measured at a wavelength of 520 nm in a UV–visible spectrophotometer (Shimadzu UV 1600 Double Beam Spectrophotometer, Kyoto, Japan). The OD output was compared to the standard sucrose absorption curve. The sucrose lysis activity of SuSy was evaluated using 1 mL of reaction mixture consisting of 200 µL Tris-HCl (pH 7.4) including MgCl_2_ 1.1 M, NAD 2.2 mM, ATP 1.2 mM, 150 µL sucrose (325 mM), and 400 µL dialyzed preparation containing 22–24 µg protein µL^−1^. Then, 100 µL of UDP (2 M) was mixed in the mixture to onset the reaction, and the mixture was put in an incubator for 30 min at 37 °C. The procedure for protein concentration detection [88] was followed. For CWI, SAI, and neutral invertase (NI) activities, measure and analysis of the procedure described by [89,90] were used per requirement. The activity of CWI, SAI, and NI was measured in a reaction mixture with a final volume of 120 μL. The reaction mixture consisted of 30 μL of 50 mM Na-citrate buffer at pH 3.7 for CWI activity assay, 30 μL of 50 mM Na-citrate buffer at pH 5.2 for SAI activity assay, and 30 μL of 50 mM potassium phosphate buffer at pH 7.6 for NI activity assay. Additionally, the reaction mixture contained 60 μL of 125 mM sucrose and 30 μL of the sample. The incubation temperature was set at 37 °C. The reactions were halted at 20 min by adding 120 μL of alkaline reagent, followed by boiling for 18 min, and then cooling on ice for 5 min. Absorbance was detected at 540 nm using UV–visible spectrophotometer (Shimadzu UV 1600 Double Beam Spectrophotometer, Kyoto, Japan).

### 4.5. RNA Extraction and RT-qPCR Expression

RNA was extracted from the immature and maturing internodal tissues of the high- and low-sucrose sugarcane clones using TRIzolR Reagent (Plant RNA Purification Reagent for plant tissue) according to the manufacturer’s instructions (Invitrogen, Shanghai, China), and the genomic DNA was removed using DNase I (TaKaRa). The isolated RNA was quantified using ND-2000 (NanoDrop Thermo Scientific, Wilmington, NC, USA), and high-quality RNA (OD260/280 = 1.9–2.4, OD260/230 ≥ 2.5, RIN ≥ 8.5, 29S: 19S ≥ 1.2, >2 μg) was cast off to construct cDNA. RT-qPCR was performed to examine the expression of sucrose metabolic genes, including SPS, SPP, SuSy, CWI, SAI, and NI, using TSINGKE biological technology (www.tsingke.net: 28-10-2022) primers (Table 1) in Light CyclerR480 II (Roche, Basel, Switzerland). Then, 1 μg RNA of each clone was used to create cDNA using the first-strand cDNA synthesis kit (Vazyme Biotech Co., Ltd., Nanjing, China) following the manufacturer’s instructions. The RT-qPCR reaction was conducted in a total volume of 20 μL, including 2 μL of cDNA (template), 0.5 μL primer mix (10 μm each of forward and reverse primers), 10.5 μL 2× ChamQ Universal SYBR qPCR master mix (Vazyme Biotech Co., Ltd.), and 6.5 μL sterile water. The amplification parameters were as follows: 1 cycle of 30 s at 95 °C, followed by 40 cycles of 5 s at 95 °C and 15 s at 60 °C, and 1 cycle of 15 s at 95 °C, 1 min at 60 °C, and 15 s at 95 °C. The sugarcane housekeeping gene, glyceraldehyde-3-phosphate dehydrogenase (GAPDH), was used as the internal reference gene to normalize the expression level. Triplicate (n = 3) biological and technical replicates were used for each sample. The data analysis was performed using Roche’s Light Cycler R 480 version 1.5.1. The relative fold change in the expression of the gene was calculated using the 2^−△△Ct^ algorithm [91].

### 4.6. Statistical Analysis

The data acquired from examined variables were statistically analyzed using Statistix 8.1 software. The data were subjected to a two-way ANOVA, with clones as factor 1 and internodes as factor 2. The LSD test was applied to compare the significant difference, at *p* < 0.05, among both internode types and sister clones. Furthermore, figures were created by using GraphPad Prism (Version 10).

## 5. Conclusions

The main product of photosynthesis is sucrose, which is produced in sugarcane leaves and accumulates in the internodes. Its metabolism is a constant hydrolysis and resynthesis process to support respiration and store excess sucrose in the stalks.

The present investigation discovered variations in sucrose and sugar concentrations between two sister clones with high and low sucrose accumulation, GXB9 and B9. The sucrose and hexose levels varied between the immature and maturing internodes of the same clone. The clone GXB9 with high sucrose storage showed reduced sucrose content in immature internodes compared to maturing ones. In immature internodes, both clones showed higher fructose and glucose levels than in maturing internodes. Immature internodes exhibited minimal sucrose content, whereas maturing internodes demonstrated elevated sucrose levels in the low-sucrose-accumulating clone B9. The concentrations of glucose and fructose were higher in immature internodes compared to maturing internodes in B9. The sucrose concentration was higher in maturing internodes, while glucose and fructose were more abundant in immature internodes in both clones. The high-sucrose-accumulating clone exhibited a higher total sucrose content than the low-sucrose-accumulating clone.

A comparison of SPS, SPP, and SuSy activities revealed that the high-sucrose sugarcane clone exhibited elevated SPS and SPP activities. The SPS and SPP activities in maturing internodes were greater than those in immature internodes for the high-sucrose sugarcane clone. The activity of SuSy was greater in the low-sucrose clone compared to the high-sucrose clone. Additionally, the SuSy activity was higher in immature internodes compared to maturing internodes for the low-sucrose-storing clone. Susy exhibited increased activity in immature internodes of both high- and low-sucrose clones, while SPS and SPP had higher activities in maturing internodes.

The up-regulation of *SPS* and *SPP* was only found in the high-sucrose clone but not in the low-sucrose clone. *SPS* was significantly up-regulated in the maturing internodes compared with immature internodes of the high-sucrose clone. *SuSy* was significantly up-regulated in immature internodes of both clones. Hence, the significant up-regulation of *SPS* in maturing internodes presents the predominant role in the high sucrose accumulation in the high-sucrose clone. Both clones have a similar genetic background, but considerable variations in sucrose content make them appealing material for further study.

**Figure 5:**
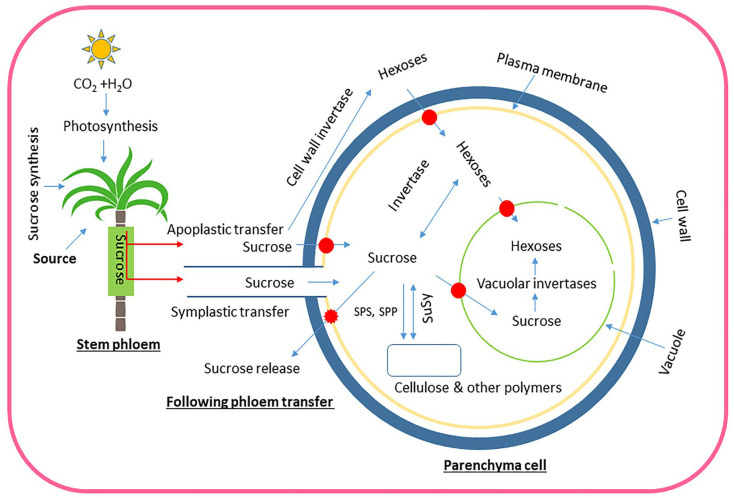
Schematic illustration depicting the process of sucrose transportation and metabolism in sugarcane, starting with its production in source leaves and ending with its storage in stems. Red arrows show sucrose transport, whereas its subsequent breakdown and storage are represented by thin blue arrows. Red ovals with blue arrows show transporters, a large dark blue circle indicates the cell wall, and the vacuole is depicted in light green. The plasma membrane is gold in color. Sucrose produced in photosynthetic leaves is transported through the phloem to stem parenchyma cells. From there, it can be transferred in two ways: symplastic (via plasmodesmata, believed to be the main route in mature sugarcane internodes) and/or apoplastic (through the cell wall space, which was considered a potential factor in earlier stages of sugarcane stem development). Sucrose may be transported to storage parenchyma by either pathway; however, apoplastic transfer may need sucrose to be broken down into hexoses by cell wall invertase. Hexoses and sucrose both enter parenchyma cells via transporters. Neutral invertases in the cytoplasm or vacuolar acid invertases may produce hexoses from sucrose inside cells. Sucrose is stored in vacuoles and cell wall space, with the equilibrium between them regulated by transporters and sucrose release into the apoplast.

The regulatory network governing sucrose metabolism in sugarcane internodes (Figure 5) still needs comprehensive elucidation. Further spatiotemporal analysis of internodes would lead to a better understanding of the roles of SPS, SPP, and SuSy in sugarcane, despite the existing knowledge about these enzymes and their involvement in sucrose metabolism.

Therefore, an integrated transcriptomic and proteomic spatiotemporal comprehensive elucidation of the sucrose metabolism mechanism in sugarcane stalks of the two sister clones should be considered in order to evaluate possible mutation in the GXB9 clone. The finding could pave the way for genetic modifications of sugarcane to increase sucrose content.

## Figures and Tables

**Table 1:** Primer sequences used in RT-qPCR amplification.

Genes Name	Forward/Reverse	5′-3′ Sequence	Product Size (bp)
*Glyceraldehyde 3-phosphate* *dehydrogenase (GAPDH)*	F	CTCTGCCCCAAGCAAAGATG	100
	R	TGTTGTGCAGCTAGCATTGGA	
*Sucrose phosphate synthase (SPS)*	F	CCATCTGTATGTTGCTGTGTGC	99
	R	GTCGGTGTCGCCCTTGTC	
*Sucrose synthase (SuSy)*	F	TGAAAATGGGATACTTAAGAAATGG	92
	R	ATAACGAACCAATGATGATATTCACCTC	
*Cell wall invertase (CWI)*	F	TCTGTACAAGCCAACCTTCG	104
	R	CCGCTTGAAATGTCAATGTC	
*Sucrose phosphate phosphatase (SPP)*	F	GGCTTTGTGCTAACCCACAT	98
	R	TTACGCACCAAATCCTCTCC	
*Soluble acid invertase (SAI)*	F	TCCTTGCTTGCCTCTCAAAT	97
	R	ACAAATGTAGCCCTGCCTTG	
*Neutral invertase (NI)*	F	ATAAACAGCCGCACCAATTC	112
	R	GCCTCTGAGGTGGAGTCTTG	

## Data Availability

All the necessary data are included in the manuscript.
